# Identification and validation of potential reference gene for effective dsRNA knockdown analysis in *Chilo partellus*

**DOI:** 10.1038/s41598-019-49810-w

**Published:** 2019-09-20

**Authors:** Olawale Samuel Adeyinka, Bushra Tabassum, Idrees Ahmad Nasir, Iqra Yousaf, Imtiaz Ahmad Sajid, Khurram Shehzad, Anicet Batcho, Tayyab Husnain

**Affiliations:** 1 Centre of Excellence in Molecular Biology, University of the Punjab, Lahore, 53700 Pakistan; 2 SAHARA Medical College, Narowal, Pakistan

**Keywords:** RNAi, RNAi

## Abstract

*Chilo partellus* is an invasive polyphagous pest that has not been effectively managed with chemical pesticides. To select potential dsRNAs for use in an alternate control strategy, it is crucial to identify and evaluate stable reference genes for knockdown expression studies. This study evaluates the expression stability of seven candidate reference genes in *C. partellus* larvae fed on crude bacterially-expressed dsRNAs and purified dsRNAs at different time intervals, as well as the developmental stages and sexes. The expression stabilities of the reference genes were evaluated with different software programmes, such as BestKeeper, NormFinder, deltaCt, geNorm, and RefFinder. The overall results rank *ELF* as the most stably expressed reference gene when larvae were fed with crude bacteria-induced dsRNAs and purified dsRNA. However, *Tubulin* and *HSP70* were more stable under different developmental stages and sexes. The expression levels of larvae that were fed crude bacteria-induced dsRNAs of *Chitinase* and *Acetylcholinesterase* were normalized with the four most stable reference genes (*ELF*, *HSP70*, *V-ATPase* and *Tubulin*) and the least stable reference gene (*18S* and *HSP70*) based on the geNorm algorithm. The least stable reference gene showed inconsistent knockdown expression, thereby confirming that the validation of a suitable reference gene is crucial to improve assay accuracy for dsRNA-targeted gene selection in *C. partellus*.

## Introduction

*Chilo partellus (*Swinhoe) (Lepidoptera: Pyralidae), commonly known as spotted stem borer (SSB), is an invasive and polyphagous pest that hampers maize production worldwide^1,2^. The infected plants have stunted stems, impaired photosynthetic capacity, and broken tassels and stems^3^, leading to low fodder and grain production. The recent change in climate has a high tendency to accelerate its proliferation and severity of damage^4^, as predicted by several models^5,6^. The first instar larvae of *C. partellus* emerge from eggs and immediately enter the stem to feed. Therefore, they hinder the effectiveness of chemical insecticides and result in chemical abuse by farmers, especially in developing countries. The extensive use of chemical pesticides and emerging insecticide resistance necessitate the development of environmentally favourable pest management strategies. Recently, scientists have been investigating RNA interference technology as alternative control measures for agricultural pests^7,8^. However, success in Lepidoptera varies, mostly owing to differences in the processes of dsRNA uptake and the spread of the RNAi signal, as well as high levels of nuclease. A key challenge for designing RNAi-based crop protection strategies is the identification of the effective target for specific insect pests and call for screening of numerous dsRNA targets. However, most RNAi studies on agricultural pests failed to validate the best reference gene for effective evaluation of silencing effects. Gene expression profiling through RT-qPCR has become a widely used methodology to generate and interpret the data with accuracy and reproducibility. RT-qPCR has been extensively used to characterize messenger RNA (mRNA) transcripts due to its high specificity, reproducibility, and sensitivity^9^. The successful implementation of a gene knockdown strategy for the control of *C. partellus* requires the standardization of RT-qPCR for effective evaluation of the knockdown effect RNAi experiment. However, there is insufficient genomic information in *C. partellus* to identify a reference gene that has stable expression under the translational inhibitory effect of dsRNA across various experimental conditions and developmental stages in *Chilo partellus*.

There is no single or universal reference gene because several factors, such as the organism type, developmental stages, and various experimental conditions, affect the expression of reference genes. To increase the credibility of RT-qPCR data interpretation, effective normalization is required to nullify background variation and biases that might influence the expression of transcript^10,11^ due to differential expression of reference genes under specific conditions^12,13^. Most often, RT-qPCR normalization is performed without normalization, but several reference gene validation studies have been conducted for many insect species^14,15^. Different categories of reference genes were reported to be more stable across dsRNA treatment, starvation treatment, tissues and developmental stages *in Halyomorphahalys*^16^. Of the six candidate reference genes analysed in *Phenacoccus solenopsis*, *β-Tubulin* was found to be the most stable reference gene in most tested conditions^17^. *16S, RPS18* and *RPL13* were recommended for normalization of RT-qPCR data in *L. erysimi*^18^. Such variation in insect reference genes across different experimental necessitates reference gene validation depending on the experiment. To date, there is no study describing reference gene validation in *Chilo partellus*, especially under different types of dsRNA treatment.

In our study, we evaluated the stability of seven reference genes, *18S* (*18S ribosomal RNA*), *V-ATPase*, *Actin*(*beta-Actin*), *ELF* (*Elongation factor 1 alpha*), *RPL32 (ribosomal protein L32*), *Tubulin* (*beta*-*Tubulin*) and *HSP70* (*Heat shock protein 70*) (Table 1), representing separate gene families and functional classes and using five software algorithms, the comparative deltaCt method^19^, BestKeeper^20^, geNorm^21^, NormFinder^22^, and online platform-RefFinder^23^. To the best of our knowledge, this report is the first to describe reference gene validation for *C. partellus* under the influence of purified dsRNA and bacteria-induced dsRNA, as well as across developmental stages. Furthermore, we used *Chitinase* and *acetylcholinesterase*as targets of dsRNA and validated the reference genes for normalizing gene expression. Our outcome will be useful in future studies for gene expression analysis on RNAi-mediated control against this notorious pest ravaging many parts of the world.

**Table 1:** Illustration the PCR efficiency of primers used in this study.

Gene code	Gene full name	Accession number	Amplicon	Slope	R^2^	Efficiency (%)
RPL32	Ribosomal protein L32	MH430673	132	−3.275	0.995	101.970
V-ATPase	Vacuolar-ATPase	MH430675	112	−3.151	0.993	107.630
Tubulin	Beta-tubulin	MH430674	125	−3.315	0.989	100.277
HSP70	Heat shock protein 70	MH430677	114	−3.018	0.992	114.422
ELF	Eukaryotic translation elongation	MH430678	129	−3.144	0.988	107.987
18s	18S ribosomal RNA	MH430676	98	−3.477	0.980	93.911
Actin	Beta-actin	MH430679	123	−3.0633	0.991	112.053

## Results

### Candidate reference gene selection and amplification

**Figure 1:**
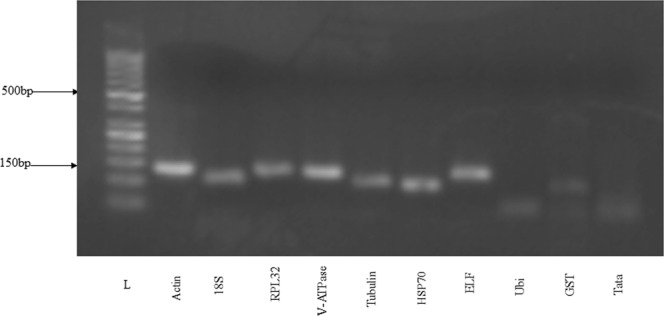
Amplification specificity of each candidate reference gene on 2.5% agarose gel, where L = 50 bp ladder.

A total of ten candidate reference genes,*18S* (*18S ribosomal RNA*), *V-ATPase*, *Actin*(*beta-Actin*), *ELF* (*Elongation factor 1 alpha*), *RPL32 (ribosomal protein L32*), *Tubulin* (beta-*Tubulin*), *HSP70* (*Heat shock protein 70*), *GST* (*Glutathione S-transferase*), *UBI* (*Ubiquitin*) and *TATA* (*TATA box-binding protein box*), were selected from the literature based on their usage as reference genes in lepidopteran. Total RNA within the range of 580 to 730 ng/µl with an adequate 260/280 absorbance ratio that signifies a lack of protein contamination proceeded to synthesize cDNA. The optimal annealing temperature of all reference genes determined from gradient PCR was 58 °C. At this temperature, single band amplification was confirmed on a 2.5% agarose gel at the expected size (Fig. 1).

**Figure 2:**
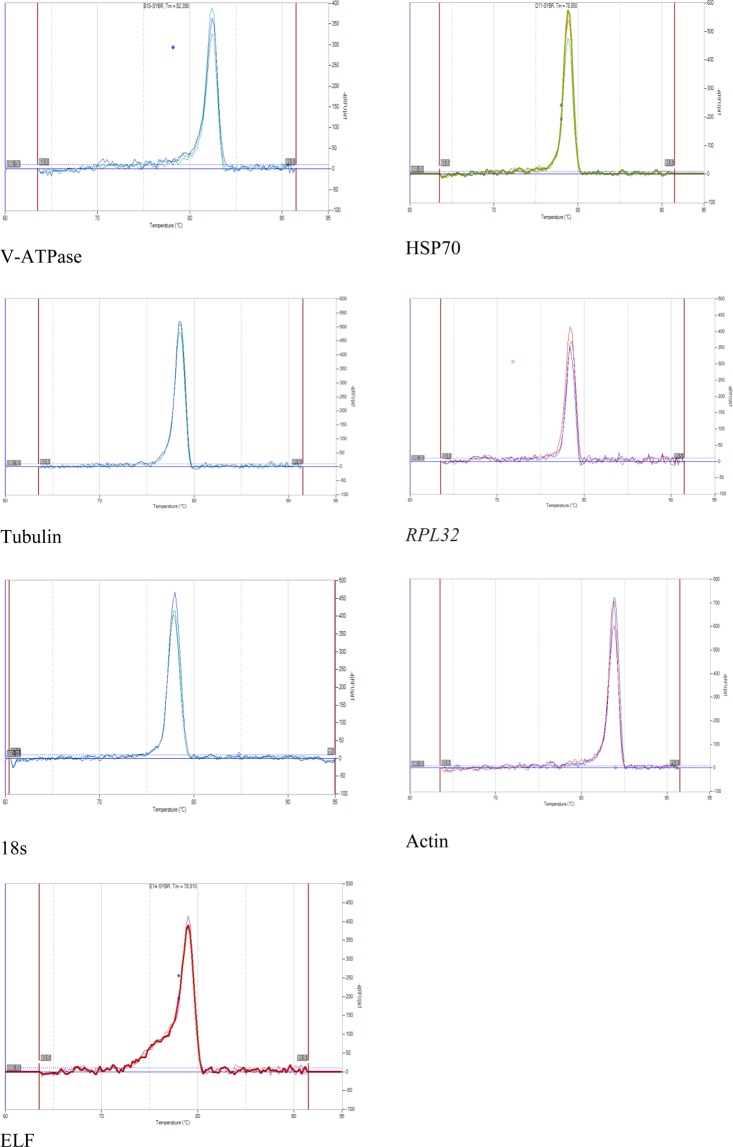
Melting curve analysis of 7 candidate reference gene with individual single peak indicating the specificity of the amplifications.

The sequence derived from amplified genes of *C. partellus* showed high homology of 87% to 95% with most lepidopteran gene sequences in the NCBI database. The sequences were submitted to NCBI and assigned with the following GenBank accession numbers: MH430673, MH430674, MH430675, MH430676, MH430677, MH430678, and MH430679. The melting curve analysis for most reference genes indicated a single peak, confirming the high specificity of individual primers (Fig. 2). However, three primers (*GST*, *UBI*, and *TATA*) that yielded unspecific peak amplifications were not employed in further analysis. A standard curve was generated from a 10-fold dilution series to evaluate the efficiency of the primers (see Supplementary Fig. S1). The correlation regression coefficient R^2^ for all primer ranges from 0.980 to 0.995, while the PCR efficiency ranges from approximately 94% to 114% (Table 1).

### Expression levels of candidate reference genes

**Figure 3:**
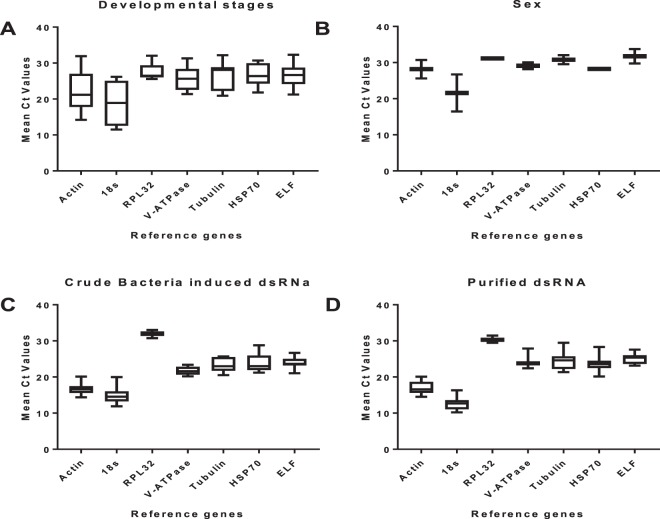
Expression profiles of candidate reference gene in *Chilo partellus*. (**A**) Developmental stage, (**B**) sex, (**C**) crude extracts of bacterially-expressed dsRNA, and (**D**) Purified dsRNA. Expression data are displayed as mean Ct values for each reference gene in all samples. Whiskers represent the maximum and minimum value while the box indicates the 25 and 75th percentiles and line across the box indicates the median.

Expression intensities of *C. partellus* transcripts were analysed after feeding on crude bacteria-induced dsRNA and purified dsRNA, as well as in different developmental stages and sexes. The Ct value in developmental stages was within 11.53–32.32, while it was 11.9–33.03 and 10.2–31.48 in larvae fed with crude bacteria-induced dsRNA and purified dsRNA, respectively. Additionally,*18S* has the lowest mean Ct value of 10.2, while RLP32 has the highest Ct value of 33.03 across all the experimental conditions. Furthermore, *18S* has the minimum average Ct (12.57 ± 0.44), while *RPL32* has the maximum average Ct (30.36 ± 0.17) in the larva group fed purified dsRNA (*18S* < *Actin < HSP70* < *VATPase* < *Tubulin* < *ELF* < *RPL32*). Likewise, the average mean Ct of those that feed on crude bacteria followed the same trend as*18S* (14.75 ± 0.70) minimum and *RPL32* (31.94 ± 0.23) maximum (Fig. 3). *18S* exhibited a high variation in developmental stage compared to sex, bacteria-induced dsRNA and purified dsRNA. The mean Ct values of *V-ATPase*, *ELF*, *tubulin* and *HSP70* showed moderate expression, while *18S* Ct was highly expressed across all conditions.

### Candidate reference gene expression stability

The Normfinder excel sheet algorithm utilized the relative quantities of Ct values for the analysis of stably expressed genes. The lower stability value of a particular reference gene corresponds to more stable expression. In larvae fed purified dsRNA, *ELF* was ranked as the most stable with lowest stability values of 0.177 and 0.33 for 5 days and 15 days, respectively. Similar results were observed in larvae fed with crude bacteria inducing dsRNA at 5 and 15 days. *Tubulin* was ranked most stable with the least stability value across developmental stage, as well as in sex differentiation (Table 2).

**Table 2:** Stability evaluation of candidate reference genes during feeding assay of *C. partellus* fed with purified and crude extracts of bacterially-expressed dsRNA over a period of 5 and 15 days using Normfinder, geNorm and BestKeeper algorithms and deltaCt algorithm.

Purified-dsRNA						Bacterially- Induced dsRNA				Developmental stages		Sex	
Algorithm	Reference gene	5days of feeding		15 days of feeding		5 days of feeding		15 days of feeding					
NormFinder		stability	Rank	stability	Rank	stability	Rank	stability	Rank	stability	Rank	stability	Rank
	Actin	0.408	4	0.562	5	0.877	4	1.188	7	2.37145	5	0.617	4
	18S	0.395	3	0.464	2	0.932	5	0.762	3	2.999018	6	3.92	6
	RPL32	0.808	6	0.529	3	1.072	7	0.87	6	1.955363	4	1.818	5
	V-ATPase	0.291	2	0.552	4	0.418	1	0.805	4	0.484509	2	0.379	3
	Tubulin	0.449	5	0.836	7	0.516	3	0.51	2	0.425675	1	0.149	1
	HSP70	0.977	7	0.807	6	1.534	6	0.827	5	1.736871	2	1.818	5
	ELF	0.177	1	0.33	1	0.481	2	0.453	1	1.738245	3	0.272	2
Bestkeeper	Actin	1.2	4	0.91	4	0.51	2	1.45	7	3.59	6	2.55	5
	18S	1.57	5	0.92	5	1.19	4	0.93	3	5.04	7	5.14	6
	RPL32	0.57	1	0.42	2	0.4	1	0.43	1	1.25	1	0.02	1
	V-ATPase	1.19	2	0.82	3	0.73	3	1.03	4	2.3	3	0.94	2
	Tubulin	1.16	3	1.08	6	1.32	6	1.08	6	2.96	4	1.24	3
	HSP70	1.98	6	1.17	7	2.39	7	1.06	5	2.9	5	0.02	1
	ELF	1.2	4	0.4	1	1.26	5	0.66	2	2.05	2	2	4
∆CT	Actin	0.94	3	1.15	4	1.69	4	1.93	7	3.76	6	2.64	4
	18S	0.97	4	1.08	2	1.8	5	1.58	5	5.01	7	5.69	7
	RPL32	1.35	6	1.12	3	1.84	6	1.66	6	3.63	5	2.82	5
	V-ATPase	0.86	2	1.17	5	1.46	1	1.53	3	2.63	2	2.14	2
	Tubulin	0.97	4	1.45	7	1.51	3	1.4	2	2.54	1	2.07	1
	HSP70	1.54	7	1.42	6	2.4	7	1.56	4	2.66	3	2.82	5
	ELF	0.8	1	1	1	1.5	2	1.31	1	3.46	4	2.25	3
Genorm	Actin	0.57	3	0.903	3	2.734	5	1.566	6	2.734	5	1.81	3
	18S	0.671	4	0.866	2	3.383	6	1.421	5	3.383	6	2.918	6
	RPL32	0.87	5	0.426	1	2.263	4	1.147	3	2.263	4	0.12	1
	V-ATPase	0.501	1	0.949	4	1.378	2	0.692	1	1.378	2	0.9	2
	Tubulin	0.501	1	1.199	6	0.92	1	1.308	4	0.92	1	1.116	4
	HSP70	1.062	6	1.1	5	0.92	1	0.692	1	0.92	1	0.12	1
	ELF	0.599	2	0.426	1	2.016	3	0.813	2	2.016	3	1.496	5

BestKeeper categorized stability based on the estimation of the Ct variation. The lower the variation is, the more stable the reference gene expression is, while the higher the variation is, the less stable the expression is. A reference gene with an average standard deviation less than one is generally acceptable to be more stable or more suitable. BestKeeper ranked *RPL32* as more stable when *C. partellus* larvae were fed purified dsRNA for 5 days and ranked *RPL32* and *ELF* as more stable when the larvae were fed purified dsRNA for 15 days (Table 2). A similar result was observed in larvae that were fed crude bacteria inducing dsRNA. In developmental stages, BestKeeper ranked *RPL32* and *ELF* as more stable, whereas *RPL32* and *HSP70* were the most suitable genes in sexes.

In deltaCt analysis, *ELF* was identified as the most stably expressed in larvae that fed on crude bacteria dsRNA after 5 days and 15 days, while *Tubulin* and *HSP70* were the least stable. *V-ATPase* and *ELF* was most expressed on the 5th and 15th days, respectively, when the larvae were challenged with purified dsRNA (Table 2). In both developmental stages and sexes, the deltaCt method ranked *Tubulin* as most stable reference gene, while *18S* was the least stable (see Supplementary S2).

**Figure 4:**
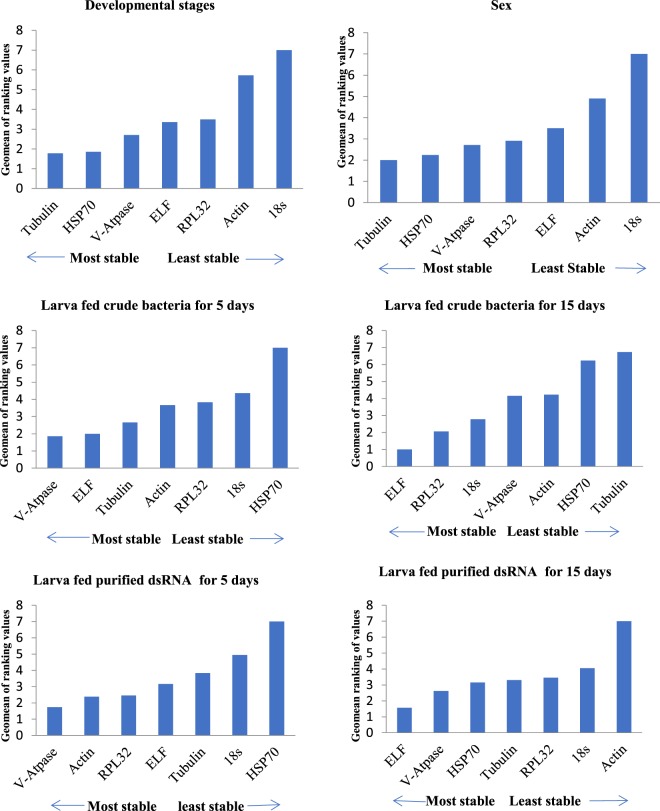
ReFinder stability ranking of 7 candidate reference genes under the influence of crude bacterially expressed and purified dsRNA fed for various time intervals and developmental stages and sex. The value with least geomean value indicated the most stable reference gene in all experimental conditions.

RefFinder makes use of all other algorithms used in the validation analysis and assigns appropriate weightage to calculate the geometric mean for each individual gene. Geomean ranking illustrated that *V-ATPase* was the most stably expressed after 5 days of feeding on crude extracts of bacterially expressed dsRNA. However, it was found that with an increase in the number of feeding days, the expression of *ELF* was the most stable. Conversely, *V-ATPase* was the most stable when the larvae were fed purified dsRNA, while *ELF* was the most stable for 15 days of feeding on purified dsRNA. *Tubulin* remained the most stable, while *Actin* and *18S* were the least stable, across all developmental stages and sexes, respectively (Fig. 4).

**Figure 5:**
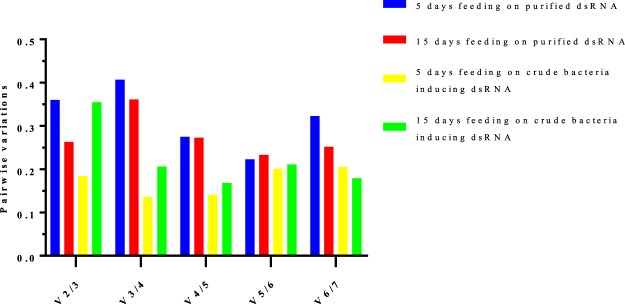
Pairwise variation (Vn/Vn + 1) analysis of the candidate reference genes to determine the optimal number of reference genes required for RT-qPCR data normalization in *Chilo partellus* by geNorm program.

geNorm analysis employs the average expression stability measure (*M*) of <1.5 to evaluate reference gene stability. The least *M* value of average expression stability corresponds to the most stably expressed reference gene. *V-ATPase*, *Tubulin* and *ELF* were identified as the most stable on the 5th day of feeding purified dsRNA, while *RPL32* and *ELF* were the most stable on the 15th day of feeding. When the larvae were fed bacterially expressed dsRNA, geNorm ranked *HSP70* and *Tubulin* as the most stable reference genes for 5 days *and HSP70* and *V-ATPase* as the most stable reference genes for 15 days (Table 3). Furthermore, pairwise variation (*V*_*n*_/*V*_*n*+ 1_) was performed to determine the optimal number of reference genes. Unfortunately, none of the reference genes evaluated was below the <0.15 general assumption cut-off value. Interpreting our data based on the trend observed, our results indicated that approximately five genes are required for normalization when *C. partellus* was fed on purified dsRNA, while four reference genes are required for normalizing the expression data for *C. partellus* feeding on bacteria-induced dsRNA (Fig. 5).

**Table 3:** Details of RT-qPCR primers.

Gene full name	Primers (5′−3′)
Ribosomal protein L32	F: AGATGGCTATAAGACCTGTT R: ACTCTGTTGTCAATACCTCT
Vacuolar-ATPase	F: CTACAGGCATGTTGGATGTGTT R: CGTGGTAACGAGATGTCTGAAG
Beta-tubulin	F: GTCGTAGAACCGTACAAC R: CGGAAGCAGATGTCATAT
Heat shock protein 70	F: AACCTACTGCTGCTGCGATT R: CACATCAAAAGTGCCACCAC
Eukaryotic translation elongation	F: AGGAAATCAAGAAGGAAGTATCC R: CAAGGCATTTTGGTTGAAGG
18S ribosomal RNA	F: CAGAACTCCGAGGTAATGATT R: CAAATGCTTTCGCTGATGTT
Beta-actin	F: GTTGACATCCGTAAGGACCTGT R: GATGATCTTGATCTCGATGGTG
Acetylcholinesterase	F: TCAGCAGTCAATAGAGAAC R: GGAATCTTACAGTCTGGTA
Chitinase	F: TCGTAATAAGCCCAGAATC R: GTAACAATAACTACGGACTC

### Validation of the identified reference genes

**Figure 6:**
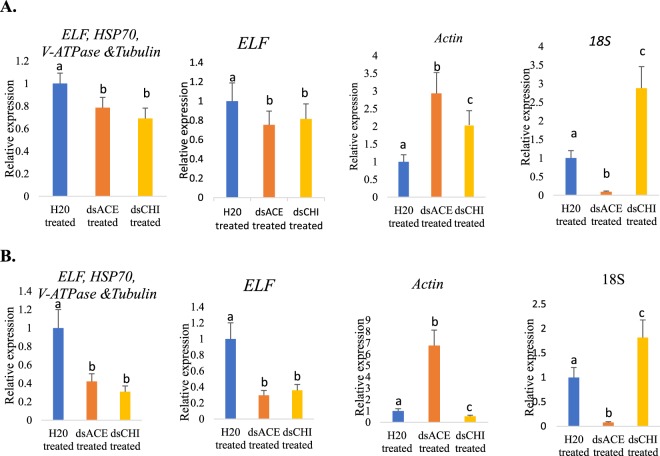
Relative expression knockdown in levels of *Chitinase* and *Acetylcholinesterase* normalized with optimum reference genes (*ELF, HSP70, V-ATPase* and *Tubulin*), *ELF* (as most stable according to NormFinder, deltaCt, RefFinder algorithm), and two least stable reference gene (*Actin* and *18S*) after feeding *Chilo partellus* larvae for (**A)** 5 days of crude extracts of bacterially expressed dsRNA, (**B)** 15 days of crude extracts of bacterially expressed dsRNA. Values are expression mean ± standard error and diffirent letter indicate significant different (p < 0.05) between the two genes and control (without dsRNA treatment).

To validate the best reference gene, the transcript expression of Chitinase and Acetylcholinesterase in *C. partellus* larvae exposed to bacterially-expressed dsRNA for 5 days and 15 days was analysed by RT-qPCR. Larvae fed on diet overlaid with nuclease free water instead of dsRNA was taken as control. The transcript expression was normalized with the four stable reference genes (*ELF*, *HSP70*, *V-ATPase* and *Tubulin*) and two least stable reference genes (*Actin* and *18S*), as validated by the geNorm algorithm (Fig. 6). Interestingly, after 5 days of exposure to the dsRNA, normalization with the optimal references (*ELF*, *HSP70*, *V-ATPase* and *Tubulin*) indicated similar knockdown of expression; however, an inconsistency was observed when *Actin* and *18S* were used for normalization. Overexpression of *Chitinase* and extreme knockdown were observed with *Actin* and*18S*, respectively. Both poor results obtained with reference genes indicated overexpression of *acetylcholinesterase*. A similar inconsistency in expression was observed when the larvae were further fed dsRNA for 15 days.

## Discussion

RT-qPCR is an important technique that in comparison with other quantitative methods, such as Northern blotting, *in-situ* hybridization and cDNA arrays, has been generally used to evaluate mRNA transcript levels for particular genes. The application of accurate reference genes is crucial for valid gene expression quantification because several factors, such as organism type, developmental stage, and various experimental conditions, affect the expression of reference genes. Endogenous genes that are involved in various biological functions, including the translation of mRNA in proteins, cytoskeletal structural proteins, enzymatic delivery of aminoacyl tRNAs to ribosomes, protein folding, stress tolerance, receptor-mediated endocytosis and intracellular trafficking of lysosomal enzymes, are mostly employed as reference genes. Several reports have shown that housekeeping gene expression levels differ under different experimental conditions^15,24,25^. These results necessitate the identification of reference genes that are stably expressed under the given experimental conditions during expression analysis. Several validation studies for reference genes have been reported in many insects, as well as for agricultural pests^26,27^. Recently, the application of RNAi technology as an alternative for pest control has been increasing, but most studies failed to validate their reference genes before the evaluation of knockdown expression. *Chilo partellus* is an invasive agricultural pest that lacks adequate genomic information. Validation studies have not been reported in *Chilo partellus*,which poses a serious setback for gene expression studies. The main concern of the paper was to identify and validate the optimal reference gene that can be used for the normalization of dsRNA fed to *Chilo partellus* larvae. In this study, the stability of seven reference genes in *Chilo partellus* developmental stages and sexes, as well as larvae fed a diet supplemented with purified dsRNA and crude bacteria-induced dsRNA, were investigated using geNorm, NormFinder, BestKeeper, ΔCt method, and RefFinder.

Our analyses demonstrated that 18S was mostly expressed among other reference genes that were investigated with minimum average Ct values across developmental stages, sexes, bacteria-induced dsRNA feeding and purified dsRNA feeding. Conversely, RPL32 has the maximum mean Ct value expression, while the expression of other reference genes varied. The expression pattern of all reference genes follows the same trend in developmental stages, sex, bacteria-induced dsRNA feeding, and purified dsRNA feeding. Therefore, it is important to evaluate the best reference that has the most stable expression when *C. partellus* organisms are under dsRNA treatment because larvae grow with time, and several metabolic activities take place during developmental stages that influence gene expression. The amplification efficiencies and correlation coefficients of all of the candidate reference genes were within the accepted range^28,29^. We have been able to amplify conserved regions of some reference genes that show significantly high homology to other lepidoptera and have been deposited in the NCBI repository (Supplement 3).

The results of the *Normfinder* algorithm indicate that *ELF* is the most stable reference gene when *C. partellus* larvae were fed purified dsRNA and bacterially expressed dsRNA for 5 days for 15 days. The algorithm further identifies *Tubulin* as the most stable across all developmental stages. The results are consistent with^17^, where *Tubulin* was found to be highly stable in *Phenacoccus solenopsis* at developmental stages. Similar to the results obtained with the *NormFinder*, delta*Ct* analysis identified *ELF* as the most stable expression across the same conditions. However, with the *Bestkepeer* algorithm, *RPL32* was identified as the most stable in most conditions, except on the 15th day feeding of pure dsRNA, where *ELF* was more stable.

*RefFinder* geomean analysis showed that *ELF* and *V-ATPase* were the most stable in both pure dsRNA and bacterially fed larvae at different time intervals. Interestingly, when comparing all algorithms for developmental stages, *Tubulin* was identified as the most stable best reference in most algorithms, except *Bestkeeper*. However, we found that *Actin and18S* are not suitable reference genes for studies that involve developmental stage and sexes in *C. partellus*. Although, *Actin and 18S* are commonly used in RT-qPCR analysis, our study ranked them as the least stable genes in most of the experimental condition. Our outcome is consistent with some previous studies in which *Actin* and *18S* were considered not suitable reference genes under the given experimental conditions^17,30–32^. This result further underscored the importance of performing validation experiments, as *Actin* and *18S*, which are highly expressed in *C. partellus*, do not provide any significant stability under this feeding experiment and developmental stages.

The *geNorm* elimination approach is based on cumulative standard deviation. This approach calculates an expression stability value (M) for each gene and compares the pair-wise variation (V) of this gene with the others to determine the optimal number of reference genes required for accurate normalization^21^. Although a value less than 0.15 is generally used to propose the maximum number of reference genes required for pairwise variation analyses, the trend of the result is also informative, as 0.15 is not to be taken as an overly strict cut-off. However, normalization with the 3 best reference genes in most cases produces more accurate and reliable normalization compared to the use of only one single reference gene. Our data indicated that five reference genes are important for validation when purified dsRNA are fed to *C. partellus*, while four best reference genes are needed when feeding assays include crude extracts of bacterially expressed dsRNA. This discrepancy might be due to the stability of dsRNA because naked dsRNA is generally less stable than bacterially expressed dsRNA.

We further compared the expression of the four optimal reference genes with ELF alone. Most algorithms rank this reference gene (ELF) as most stable under dsRNA influence irrespective of exposure time. Interestingly, normalization of the two-targeted genes with four optimal reference genes and ELF only yielded similar results. Based on this result, it was concluded that *ELF* can be used for effective normalization and evaluation of the dsRNA silencing effect in this species. ELF encodes a protein that is involved in protein synthesis and has been validated as a potential candidate for expression studies in many insects^25,33,34^. Furthermore, Tubulin should be used when the experimental objective involved developmental stages and sexes of *C. partellus*. Tubulin is an cytoskeletal structure proteins and its stability has been shown to varies under different experimental conditions in insect^35–39^.Acetylcholinesterase is an indispensable enzyme in insect central nervous system that terminates nerve impulse transmission at synaptic junctions of cholinergic neurons through neurotransmitter acetylcholine hydrolyzation. Whereas insect chitinase plays crucial role in chitin digestion and in the insect gut peritrophic membrane^40^ where it facilitates digestion and protects against micro-organism invasion. Silencing of both genes are documented in several insects^41–46^ without any prior reference gene validation. Our result highlights the importance of reference gene normalization to avoid false discernment of target gene expression. Thus, we suggest that to prevent incorrect interpretation and incorrect evaluation of the knockdown effect, proper reference gene validation should be performed prior to expression studies. This study establishes a foundation for future research that might involve RT-qPCR because it is the first study investigating reference gene normalization in *C. partellus*.

## Materials and Methods

### Insect rearing and feeding on dsRNA supplemented artificial diet

Maize stem borer (*Chilo partellus*) larvae were collected from maize experimental plots and transferred to insectary facilities at the Centre of Excellence in Molecular Biology, Pakistan. The larvae were sorted according to their growth stages and maintained on fresh maize stem for approximately 3 days to adapt to the new artificial environment (26 °C ± 2 and 65% humidity). dsRNA were targeted against vital genes that have been reported to induce silencing in order Lepidoptera: *Chitinase*, *Vacuolar ATPase*, *Arginine kinase*^47^, *acetylcholinesterase*^45^, and *cytochrome P450*^48^. dsRNAs were induced in bacteria as described by^14^ and purified according to^49^. Briefly, individual amplified genes were ligated into the L4440 vector and were transformed into the HT115 *E. coli* strain and subjected to induced dsRNA synthesis by the addition of 0.6 mmol L^−1^ IPTG for 4 hours. The dsRNA-inducing bacterial culture was pelleted down and resuspended in 10 mM EDTA 1 M ammonium acetate to a 1/20 initial induction volume. Then, an equal volume of phenol:chloroform:isoamyl alcohol (25:24:1) pH 8.0 was added, and the samples were vortexed vigorously. The samples were incubated at 65 °C for 30 min and centrifuged at 10,000 *g* for 20 min. The cleared upper phase was transferred to new 50 ml tubes containing equal amounts of isopropanol and incubated at −20 °C overnight. The nucleic acid precipitates were pelleted down at 10,000 *rpm* for 20 min and immediately treated with 0.4 U/µl DNase and 0.2 µg/µl RNase A for 30 min. The larvae were fed an artificial diet^50^ with some modification. To monitor developmental growth, L5 larvae were maintained until the next L5 generation. Samples were collected as the L5 metamorphose to pupa, adult, egg batches, as well as L1, L2, L3, L4, L5. All collected samples were ground in Trizol reagent (Thermo Scientific) and stored at −70 °C. For dsRNA feeding, L3 stage larvae were selected and divided into two groups comprising 20 insects per dsRNA treatment: group I was fed an artificial diet coated with bacterially-expressed dsRNA resuspended in nuclease-free water, while group II was fed on artificial diet supplemented with purified dsRNA. Larvae fed a diet supplemented with ordinary nuclease-free water were used as controls. Three biological replicate samples of each dsRNA fed larvae were ground in Trizol reagent (Thermo Scientific) and stored at −70 °C.

### RNA Isolation and cDNA preparation

Total RNA was extracted from grinded samples stored in Trizol Reagent (Sigma-Aldrich, St. Louis, USA) according to the manufacturer’s instructions. Potential DNA impurities were removed by treating the total RNA with DNase I (#EN0521). A Nanodrop ND-1000 spectrophotometer (Nanodrop Technologies) was used to determine the quality and purity of the RNA. RNA samples with A260/A280 ratios between 1.9 and 2.1 and A260/A230 ratios higher than 2.0 were used in the analysis. One microgram of the total RNA for each sample was reverse transcribed to cDNA with Oligo dT primers using the RevertAid first Strand cDNA synthesis kit (#K1622) according to the manual instructions.

### Reference genes selection and primer design

Ten candidate reference genes, namely,*18S* (*18S ribosomal RNA*), *V-ATPase*, *Actin*, *ELF*, *RPL32*, *Tubulin* and *HSP70*, *Glutathione S-transferase*, *Ubiquitin* and *TATA* box-binding protein (TBP) box, were selected from the literature based on frequent usage for normalization in agricultural pests. *C. partellus* lacks adequate nucleotide information in the database; therefore, we designed primers for the maximum conserved region from order Lepidoptera found in NCBI databases. Primers within the range of 80 bp to 200 bp (Table 1) were designed with online primer 3plus software (www.bioinformatics.nl/primer3plus) default setting, except that the minimum GC% was adjusted to 45 and the maximum to 60. The 3′ complementary, potential hairpin formation and self-annealing site were evaluated with the online Oligonucleotide property calculator^51^ (http://biotools.nubic.northwestern.edu/OligoCalc.html). Finally, the primer sequences were blasted on the NCBI database to evaluate the probability of individual gene-specific hits. The primer oligonucleotides were synthesized from Eurofins Genomics, Louisville, USA (Table 3).

### Primers competent evaluation for RealTime-quantitative PCR

The annealing temperature for reference genes was optimized by gradient PCR, and the amplified fragments were sequenced through the facility at CEMB (Pakistan) and submitted to NCBI GenBank. RT-qPCR was performed in a Piko real 96 Real-time PCR system to evaluate the peak specificity and the standard curve data using a triplicate 10-µl reaction. The reaction mixture (10 µl) contained Maxima SYBR Green RT-qPCR 2X Master mix (Thermo scientific), 500 nM of each forward and reverse primer, cDNA and nuclease-free water. Amplifications were performed according to the following cycling profile: an initial denaturation at 95 °C for 5 min followed by 35 cycles of denaturation at 95 °C for 30 s, annealing at 58 °C for 30 s, and extension at 72 °C for 30 s. Temperature from 60 °C to 90 °C was added at the end of the run to check the PCR specificity. The Ct values against the semi-log of dilution series were used to generate a standard curve, and the respective slope was inserted into the formula E (%) = (10^−*1/slope*^ − 1) × 100% for the evaluation of amplification efficiency (E) for each specific primer.

### Statistical analysis

The average Ct values of three technical replicates from pooled cDNA of three biological replicates were used to evaluate the stability of the candidate reference. The stability of the candidate reference genes was evaluated using the *deltaCt* method^19^, *BestKeeper*^20^, *geNorm*^21^, *NormFinder*^22^, and online platform-*RefFinder*^23^. *RefFinder* provides comprehensive ranking based on geometric mean and integrates the other computational algorithm.

### Stability assessment of selected reference genes

To evaluate the reference gene stability, larvae were fed bacterially expressed *Chitinase* dsRNA and *acetylcholinesterase* dsRNA for 5 days and 15 days. Larvae fed on artificial diet overlaid with nuclease free water instead of dsRNA was used as control. RT-qPCR reactions were performed in biological and technical triplicates. The transcript expression was normalized with the four stable reference genes (*ELF*, *HSP70*, *V-ATPase* and *Tubulin*) and two least stable reference genes (*Actin* and *18S*), as validated by the geNorm algorithm. RT-qPCR was performed as described above, and gene expression analysis was performed using the Livak method^52^. One-way ANOVA was used to analyse the significance for gene expression, and the mean gene expression was compared by Tukey’s multiple comparison (*P* < 0.05) using GraphPad Prism software.

## Supplementary information

Supplementary information

## References

[1] Kfir R, Overholt WA, Khan ZR, Polaszek A (2002). Biology and management of economically important lepidopteran cereal stem borers in Africa. Annu. Rev. Entomol..

[2] Farid A (2007). Studies on maize stem borer, Chilo partellus in Peshawar valley. Pak. J. Zool..

[3] Bosque-Pérez NA, Mareck JH (1990). Distribution and species composition of lepidopterous maize borers in southern Nigeria. Bull. Entomol. Res..

[4] Adeyinka OS (2018). A lag in the advancement of biotechnology: Reliable control of maize stem borers in. Africa. J. Plant Prot. Res..

[5] Khadioli N (2014). Effect of temperature on the phenology of Chilo partellus (Swinhoe) (Lepidoptera, Crambidae); simulation and visualization of the potential future distribution of C. partellus in Africa under warmer temperatures through the development of life-table param. Bull. Entomol. Res..

[6] Tamiru A, Getu E, Jembere B, Bruce T (2012). Effect of temperature and relative humidity on the development and fecundity of Chilo partellus (Swinhoe) (Lepidoptera: Crambidae). Bull. Entomol. Res..

[7] Burand JP, Hunter WB (2013). RNAi: future in insect management. J. Invertebr. Pathol..

[8] Bolognesi R (2012). Characterizing the Mechanism of Action of Double-Stranded RNA Activity against Western Corn Rootworm (Diabrotica virgifera virgifera LeConte). PLoS One.

[9] Bustin SA (2000). Absolute quantification of mRNA using real-time reverse transcription polymerase chain reaction assays. J. Mol. Endocrinol..

[10] Bustin SA (2002). Quantification of mRNA using real-time reverse transcription PCR (RT-PCR): trends and problems. J. Mol. Endocrinol..

[11] Huggett J, Dheda K, Bustin S, Zumla A (2005). Real-time RT-PCR normalisation; strategies and considerations. Genes Immun..

[12] Shi C (2016). Evaluation of Housekeeping Genes for Quantitative Real-Time PCR Analysis of Bradysia odoriphaga (Diptera: Sciaridae). Int. J. Mol. Sci..

[13] Suzuki T, Higgins PJ, Crawford DR (2000). Control Selection for RNA Quantitation. Biotechniques.

[14] Vatanparast M, Kim Y (2017). Optimization of recombinant bacteria expressing dsRNA to enhance insecticidal activity against a lepidopteran insect, Spodoptera exigua. PLoS One.

[15] Chang Y-W (2017). Selection and validation of reference genes for quantitative real-time PCR analysis under different experimental conditions in the leafminer Liriomyza trifolii (Diptera: Agromyzidae). PLoS One.

[16] Bansal R (2016). Quantitative RT-PCR Gene Evaluation and RNA Interference in the Brown Marmorated Stink Bug. PLoS One.

[17] Arya SK (2017). Reference genes validation in Phenacoccus solenopsis under various biotic and abiotic stress conditions. Sci. Rep..

[18] Koramutla MK, Aminedi R, Bhattacharya R (2016). Comprehensive evaluation of candidate reference genes for qRT-PCR studies of gene expression in mustard aphid, Lipaphis erysimi (Kalt). Sci. Rep..

[19] Nicholas, S., Steve, B., Jie, J. & Swee, L. T. Selection of housekeeping genes for gene expression studies in human reticulocytes using real-time PCR. *BMC Mol. Biol*. **7**,(2006). 10.1186/1471-2199-7-33 PMC1609175 17026756

[20] Pfaffl MW, Tichopad A, Prgomet C, Neuvians TP (2004). Determination of stable housekeeping genes, differentially regulated target genes and sample integrity: BestKeeper – Excel-based tool using pair-wise correlations. Biotechnol. Lett..

[21] Vandesompele J (2002). Accurate normalization of real-time quantitative RT-PCR data by geometric averaging of multiple internal control genes. Genome Biol..

[22] Andersen CL, Jensen JL, Ørntoft TF (2004). Normalization of Real-Time Quantitative Reverse Transcription-PCR Data: A Model-Based Variance Estimation Approach to Identify Genes Suited for Normalization, Applied to Bladder and Colon Cancer Data Sets. Cancer Res..

[23] Xie F, Xiao P, Chen D, Xu L, Zhang B (2012). miRDeepFinder: a miRNA analysis tool for deep sequencing of plant small RNAs. Plant Mol. Biol..

[24] Valente, V. *et al*. Selection of suitable housekeeping genes for expression analysis in glioblastoma using quantitative RT-PCR. *Ann. Neurosci*. **21** (2014). 10.5214/ans.0972.7531.210207 PMC4117159 25206063

[25] Van Hiel MB (2009). Identification and validation of housekeeping genes in brains of the desert locust Schistocerca gregaria under different developmental conditions. BMC Mol. Biol..

[26] Yang X, Pan H, Yuan L, Zhou X (2018). Reference gene selection for RT-qPCR analysis in Harmonia axyridis, a global invasive lady beetle. Sci. Rep..

[27] Sun M, Lu M-X, Tang X-T, Du Y-Z (2015). Exploring Valid Reference Genes for Quantitative Real-Time PCR Analysis in Sesamia inferens (Lepidoptera: Noctuidae). PLoS One.

[28] Rutledge RG, Côté C (2003). Mathematics of quantitative kinetic PCR and the application of standard curves. Nucleic Acids Res..

[29] Chervoneva I, Li Y, Iglewicz B, Waldman S, Hyslop T (2007). Relative quantification based on logistic models for individual polymerase chain reactions. Stat. Med..

[30] Zheng Y-T, Li H-B, Lu M-X, Du Y-Z (2014). Evaluation and Validation of Reference Genes for qRT-PCR Normalization in Frankliniella occidentalis (Thysanoptera:Thripidae). PLoS One.

[31] Yuan M (2014). Selection and Evaluation of Potential Reference Genes for Gene Expression Analysis in the Brown Planthopper, Nilaparvata lugens (Hemiptera: Delphacidae) Using Reverse-Transcription Quantitative PCR. PLoS One.

[32] Cheng D, Zhang Z, He X, Liang G (2013). Validation of Reference Genes in Solenopsis invicta in Different Developmental Stages, Castes and Tissues. PLoS One.

[33] Ma K-S (2016). Identification and Validation of Reference Genes for the Normalization of Gene Expression Data in qRT-PCR Analysis in Aphis gossypii (Hemiptera: Aphididae). J. Insect Sci..

[34] Rajarapu SP, Mamidala P, Mittapalli O (2012). Validation of reference genes for gene expression studies in the emerald ash borer (Agrilus planipennis). Insect Sci..

[35] Yang C (2015). Selection of reference genes for RT-qPCR analysis in a predatory biological control agent, Coleomegilla maculata (Coleoptera: Coccinellidae). Sci. Rep..

[36] Shang F (2015). Reference Gene Validation for Quantitative PCR Under Various Biotic and Abiotic Stress Conditions in Toxoptera citricida (Hemiptera, Aphidiae). J. Econ. Entomol..

[37] Yu S-H (2016). Identification and evaluation of reference genes in the Chinese white wax scale insect Ericerus pela. Springerplus.

[38] Liu G (2016). Evaluation of Reference Genes for Reverse Transcription Quantitative PCR Studies of Physiological Responses in the Ghost Moth, Thitarodes armoricanus (Lepidoptera, Hepialidae). PLoS One.

[39] Dai T-M, Lü Z-C, Liu W-X, Wan F-H (2017). Selection and validation of reference genes for qRT-PCR analysis during biological invasions: The thermal adaptability of Bemisia tabaci MED. PLoS One.

[40] Kramer KJ, Muthukrishnan S (1997). Insect Chitinases: Molecular Biology and Potential Use as Biopesticides. Insect Biochem. Mol. Biol..

[41] Mamta Reddy KRK, Rajam MV (2016). Targeting chitinase gene of Helicoverpa armigera by host-induced RNA interference confers insect resistance in tobacco and tomato. Plant Mol. Biol..

[42] Cao B, Bao W, Wuriyanghan H (2017). Silencing of Target Chitinase Genes via Oral Delivery of dsRNA Caused Lethal Phenotypic Effects in Mythimna separata (Lepidoptera: Noctuidae). Appl. Biochem. Biotechnol..

[43] Ganbaatar O (2017). Knockdown of Mythimna separata chitinase genes via bacterial expression and oral delivery of RNAi effectors. BMC Biotechnol..

[44] Zhou H (2017). Silencing Chitinase Genes Increases Susceptibility of Tetranychus cinnabarinus (Boisduval) to Scopoletin. Biomed Res. Int..

[45] Ye X, Yang L, Stanley D, Li F, Fang Q (2017). Two Bombyx mori acetylcholinesterase genes influence motor control and development in different ways. Sci. Rep..

[46] Saini RP (2018). Silencing of HaAce1 gene by host-delivered artificial microRNA disrupts growth and development of Helicoverpa armigera. PLoS One.

[47] Camargo RA (2016). RNA interference as a gene silencing tool to control Tuta absoluta in tomato (Solanum lycopersicum). PeerJ.

[48] Bautista MAM, Miyata T, Miura K, Tanaka T (2009). RNA interference-mediated knockdown of a cytochrome P450, CYP6BG1, from the diamondback moth, Plutella xylostella, reduces larval resistance to permethrin. Insect Biochem. Mol. Biol..

[49] Solis CF, Santi-Rocca J, Perdomo D, Weber C, Guillén N (2009). Use of Bacterially Expressed dsRNA to Downregulate Entamoeba histolytica Gene Expression. PLoS One.

[50] Kfir R (1992). A simple artificial diet for mass rearing the stem borer Chilo partellus (Swinhoe) (Lepidoptera: Pyralidae). J. Entomol. Soc. South. Afr..

[51] Kibbe WA (2007). OligoCalc: an online oligonucleotide properties calculator. Nucleic Acids Res..

[52] Livak KJ, Schmittgen TD (2001). Analysis of Relative Gene Expression Data Using Real-Time Quantitative PCR and the 2−ΔΔCT Method. Methods.

