# Supplementary material for: Identification and validation of potential reference gene for effective dsRNA knockdown analysis in Chilo partellus
Source: Sci Rep. 2019 Sep 20;9:13629. doi: 10.1038/s41598-019-49810-w (PMC6754392; doi:10.1038/s41598-019-49810-w)
Supplement: Supplementary file 1 — Supplementary information [file 41598_2019_49810_MOESM1_ESM.pdf]

Identification and validation of potential reference gene for effective dsRNA knockdown analysis in *Chilo partellus*

Authors: Olawale Samuel Adeyinka [1], Bushra Tabassum*[1], Idrees Ahmad Nasir[1], Iqra Yousaf[1], Imtiaz Ahmad Sajid[1] Khurram Shehzad[2], Anicet Batcho[1] and Tayyab Husnain[1].

Figure S1. Standard curves of the 7 candidate reference genes

Figure S2. Stability ranking of seven candidate reference gene expressions under two different treatments of feeding assay; with crude extracts of bacterially-expressed dsRNA, and Purified

Supplementary 3. List of reference gene with assigned accession number from NCBI GenBank submitted sequences

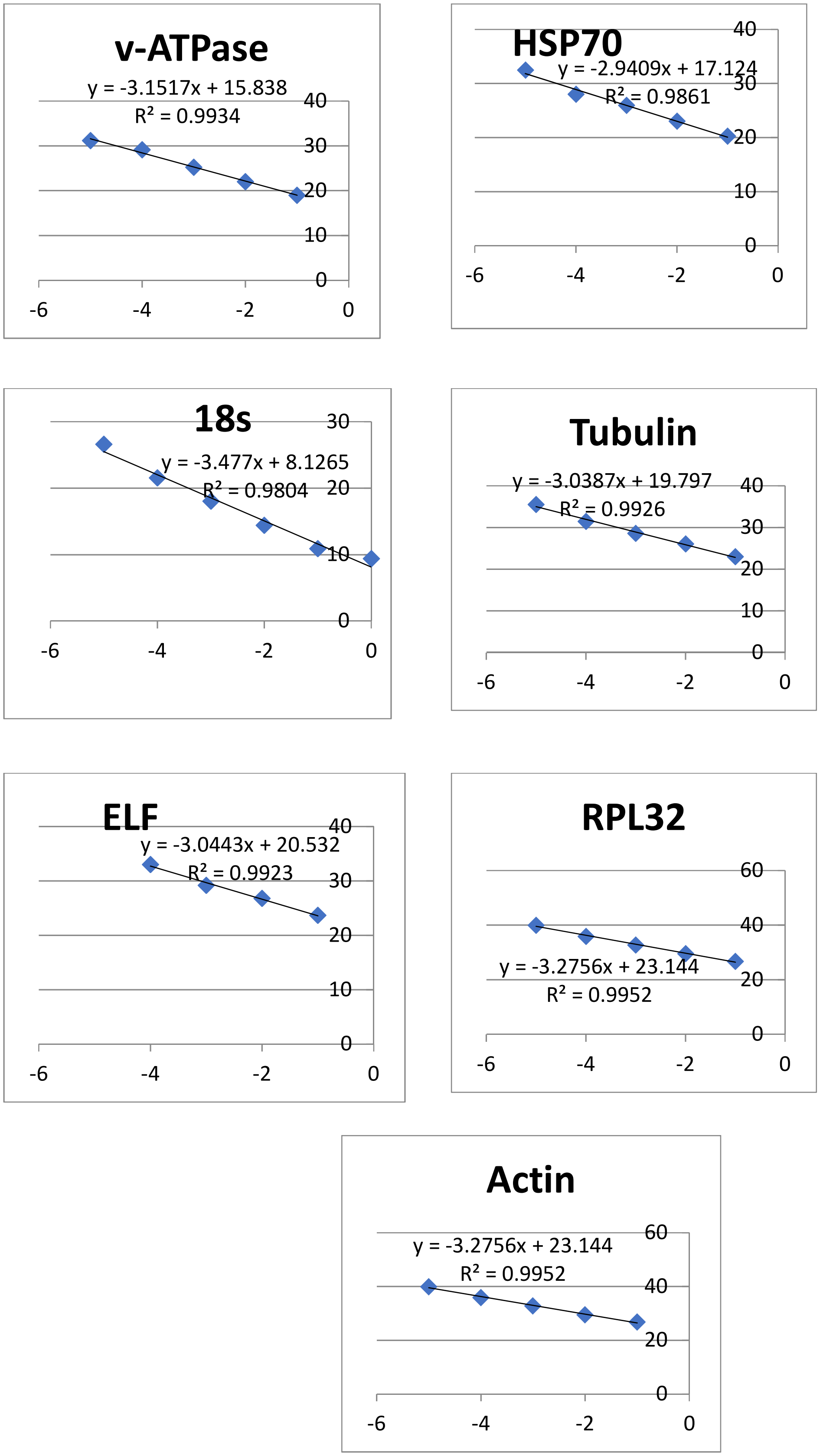

Figure S1. Standard curves of the 7 candidate reference genes

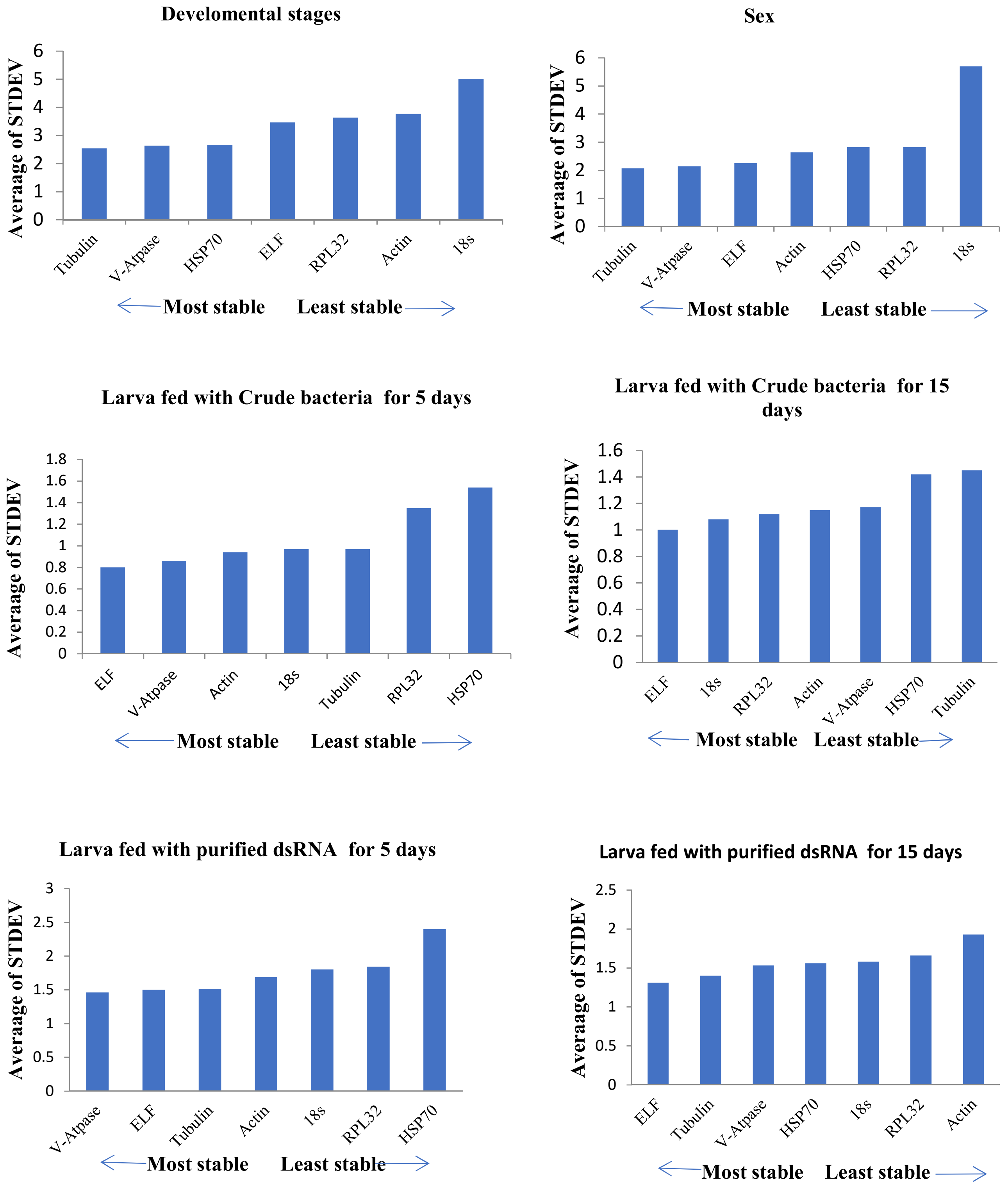

Figure S2: Stability ranking of seven candidate reference gene expressions under two different treatments of feeding assay; with crude extracts of bacterially-expressed dsRNA, and Purified dsRNA, generated by Delta CT method. Panels were represented as the stability validation results of reference genes expression in developmental stages, sex, feeding assay of 5 and 15 days on crude extracts of bacterially expressed dsRNA and purified dsRNA fractions.

Supplementary 3: List of reference gene with assigned accession number from NCBI GenBank submitted sequences

1. >MH430673 [Organism=Chilo partellus] ribosomal protein L32 (RpL32) mRNA, short sequence

CGCCCTAATTACGTCTAAAGAGGAGAAGATTTATCCGCCATCAGTCAGATCGCTATG
ACAAACTTAAGCGTAACTGGCGCAACCGAGAGGTATTGACAACAGAGTA

2. >MH430674 [Organism=Chilo partellus] beta-tubulin mRNA, short sequence

CGGGAGGGAAAATGAAAGTCGTCATAGTTTCGTCTGTGTTCTCTAGAGTCTCGGTGG
ATGACAGGAAGAGAAAAGGTTCTACGACAAAAAC

3. >MH430675[Organism=Chilo partellus] V-type ATP synthase catalytic subunit, short sequence

GCATGTTGGATGTGTGTCCACGACTCATCTTCTTCATGATGGATCCGGACGCCGTCG
AAAACCACCGACAGTTCTGGGAAAGTTTTTGTCAAACATCTCGTTACCACGAGA

4. >MH430676 [Organism=Chilo partellus] 18S ribosomal RNA gene, short sequence

ATCAAAGATCAGGGGGCTTCGTATGCGACGTTAGAGGTGTACTTCTTGGATCGTCGC
AGACGAAATCAGCGAAAGCATTGAGAAA

5. >MH430677 [Organism=Chilo partellus] heat shock protein 70 (hsp70) mRNA, short sequence

ATAACCTGCTGCTGCTGCGATTGCTTACGGTGTGGACTAGCAGTCAGGTAGTGGAGT
ACGAATGTCCTGCATTTTCGATCTCGGTGGTGGCATTTTGATGTAGAAAAAAAAAAC

6. >MH430678 [Organism=Chilo partellus] elongation factor 1 alpha mRNA, short sequence

GAACCCCCATACAGCGAATCCCGATTTGAAGAAATCAAAAAGGAAGTCTCCTCATA
CATTAAGAAAATCGGTTATAACCCAGCTGCTGTCGCTTTCGTACCTATTTCTGGTTGG
CATGGAGACAACATG

7. >MH430679 [Organism=Chilo partellus] actin mRNA, short sequence

AAAAGAGCAAGGTCGGTGTTTCTTCTGCTCCTGTCGGCGATACCGGGGTACATGGTG
GTGCAATGGACATGACGGTGTTGGCGTACAGGTCCTTACGGATGTCAACACGTCTGG
GGGTGGAAA
